# The Ca^2+^-activated Cl^−^ channel, ANO1 (TMEM16A), is a double-edged sword in cell proliferation and tumorigenesis

**DOI:** 10.1002/cam4.232

**Published:** 2014-03-18

**Authors:** Zhiqiang Qu, Weicheng Yao, Ruyong Yao, Xiangping Liu, Kuai Yu, Criss Hartzell

**Affiliations:** 1 Medical Research Center, Affiliated Hospital, Qingdao UniversityQingdao, 266555, China; 2 State Key Physiological Discipline, Qingdao UniversityQingdao, 266555, China; 3 Department of Neurosurgery, Affiliated Hospital, Qingdao UniversityQingdao, 266555, China; 4 Department of Cell Biology, School of Medicine, Emory University30322, Atlanta

**Keywords:** ANO1, Ca^2+^-activated Cl^−^ channel, cancer, cell proliferation, tumorigenesis

## Abstract

Since anoctamin 1 ANO1 (TMEM16A) was found to be a molecular component of Ca^2+^-activated Cl^−^ channels, its role in tumorigenesis has gained attention at a fast pace. ANO1 overexpression frequently occurs in the cancer tissues along with 11q13 chromosome amplification. Poor prognosis of many types of cancers has been closely correlated with *ANO1* gene amplification and protein overexpression. ANO1 is now considered an excellent biomarker for certain cancers. Recent research suggests that it is the channel function of ANO1 that is involved in the tumorigenesis. However, how the overexpression of the functional ANO1 causes malignant transformation of tissues via signaling pathways, for example, MAPK remains to be investigated. Clarification of the reasons in future will avail to make ANO1 as a target for cancer treatment.

## Introduction

Participation of ion channels or transporters in tissue tumorigenesis has not been a new topic, for example, ZIP4, a zinc transporter that has been found to be overexpressed in pancreatic cancers and significantly contributes to human pancreatic tumorigenesis 1. Nonetheless, new relevant findings have fast changed the field 2–6. Recently, anoctamin 1 (ANO1), an anion channel whose activation is strictly dependent on intracellular-free Ca^2+^ levels has been spotlighted on its roles in tumorigenesis 7–15. More and more evidence suggests that Ca^2+^ homeostasis plays important roles in the cellular events critical in tumorigenesis, that is, proliferation, metastasis, and apoptotic sensitivity, etc. 16. In many common cancer tissues or cancer cell lines, the expression or activities of Ca^2+^ channels, pumps, or transporters have been found to be either up- or downregulated dramatically, which certainly exerts effects on global cytosolic-free Ca^2+^ levels and contributes to tumorigenesis 16. Aberrant Ca^2+^ homeostasis may directly lead to tumorigenesis or through the factors whose activities are determined by the Ca^2+^ signaling. ANO1 is considered one among the factors. In this review, we have summarized the recent progresses in the study of the roles that the Ca^2+^-activated Cl^−^ channel (CaCC) ANO1 plays in the formation of tissue cancers and carcinomatous behaviors of cell lines, and discussed the potential mechanisms involved in these processes.

### ANO1

The molecular identity of the CaCC was *discovered* eventually in 2008 17–19. The identified CaCC channel, TMEM16A/ANO1, is among the 10 members of the transmembrane protein family (TMEM16 A–K or anoctamin 1–10. At least some of these proteins are anion channel proteins with eight putative transmembrane segments) 20. Since then, the field has moved to a very active status with near 200 papers published to emphasize its physiological importance in our body, such as sensory transduction, epithelial secretion, smooth muscle contraction, and cell proliferation 21–24. Knockout of ANO1 turned lethal and the mice died of severe tracheomalacia days after birth 25. Nonetheless, disease-causing mutations in ANO1 have not been reported at present. In this review, we will not talk about the edge of its physiological importance but another side of the sword: the role of ANO1 overexpression in tumorigenesis.

### ANO1 and cancer

Before it was recognized as a CaCC channel, ANO1 was found to be amplified as part of human chromosome 11q13 amplicon in cancers such as esophageal squamous cell cancer (ESCC), gastrointestinal stromal tumor (GIST), head and neck squamous cell carcinoma (HNSCC), pancreatic and breast cancers 26–29. Some investigators thought that ANO1 was a tumor marker while others an oncogene 8,15. The 1.8 Mb amplicon core in the 11q13 region was one of the most frequently amplified chromosomal regions in human cancers and correlated with a poor prognosis 7,29. Although cyclin D1 (CCND1) and fibroblast growth factor 19 (FGF19) have been considered the possible drivers of the 11q13 amplicon 30, *it remains unknown whether any gene(s) amplified from the core can malignantly transform normal tissues*.

Since the 11q13 amplification was found in different cancer types, ANO1, cloned as FLJ10621 initially 28, has been described under other names as well, for example, DOG1 (discovered on GISTs protein 1) 29, ORAOV2 (oral cancer overexpressed 2) 31, and TAOS2 (tumor-amplified and overexpressed sequence 2) 32. Although no one knows why ANO1 is amplified in tumorigenesis and how it influences cancer cell proliferation, several recent studies have provided clues to crack the mysteries 7,9. In this review, we try to pull together the past and present opinions and make a general picture for the role of ANO1 amplification and overexpression in the tumorigenesis.

## ANO1 is a Marker of Cancer Tissues

Since FLJ10261 (*ANO1*) was characterized using bioinformatics as a gene residing between FGF3 and *FADD* genes at 11q13 28, the genes including *ANO1* amplified in the 11q13 core region were considered as markers of cancers.

With an antibody to detect the immunoreactivity of DOG1 (ANO1) on the soft tissue microarrays of GIST, the DOG1 protein was found to be expressed strongly on the cell plasma membrane of all GISTs 29. Likewise, the Seethala group demonstrated that all salivary gland acinar cell carcinomas were DOG1 positive on the apical membrane around lumina while most ductal tumors were negative 8, being consistent with the function of ANO1, an anion channel on the plasma membrane. Therefore, strong staining of DOG1 as a marker helps make a diagnosis of cancer. Additionally, the Gollin group proved that the *DOG1* gene was overexpressed in both tumor tissues and tumor cell lines with or without 11q13 amplicon core amplified 32. *This evidence indicates that ANO1 overexpression in tumors is independent of the 11q13 amplification, implying that ANO1 overexpression is likely specific in tumorigenesis*.

Since the overexpression of ANO1 in HNSCC and prostate tumors could stimulate cell migration (i.e., cell movement, attachment, spreading, detachment, and invasion), which could be inhibited by ANO1 channel inhibitors 33,11, and because immunohistochemistry (IHC) revealed that ANO1 overexpression was positively correlated with lymph node metastasis of ESCC and advanced clinical stage of cancers 14, ANO1 overexpression has also been considered a potential marker for distant metastasis of cancers.

## Effect of ANO1 Overexpression and Function on the Proliferation and Migration of Carcinoma Cells

So far, ANO1 overexpression has been widely considered capable of enhancing proliferation and migration of cancer cells. However, the points of view obtained from the cancer cell lines on this issue are not unanimous (Table 1).

**Table 1:** ANO1 expression in various cancer cell lines

Cancer cell lines	Cancer tissues	ANO1 OE	11q13 amplification	ANOl OE-induced		Reference

				Proliferation	Migration	
KYSE30	ESCC	Yes		Yes		48
KYSE510		Yes		Yes		48
MKN28	GIST	Yes		Yes		20
GIST-T1		Yes		No		50
GIST882		Yes		No		50
UM-SCC1	HNSCC		Yes	Yes		14
HEp-2		No			Yes	2
SCC-25		Yes			Yes	2
BBCY		Yes		No	Yes	44
CAL-33		No		No	Yes	44
CFPAC-1	Pancreas	Yes		Yes		52
ZR.75-1	Breast	Yes	Yes	Yes		4
HCC1954		Yes	Yes	Yes		4
MDA-MB-415		Yes	Yes	Yes		4
LNCaP	Prostate	Yes		Yes	Yes	34
PC-3		Yes		Yes	Yes	34
T24	Bladder		No	Yes		14
HEK293	Kidney	No		No	No	55
HEK293T		No		Yes		12

Blank areas in columns except cancer tissues indicate unknown. OE, overexpression; ESCC, esophageal squamous cell cancer; GIST, gastrointestinal stromal tumor; HNSCC, head and neck squamous cell carcinoma.

***Viewpoint 1*****:** ANO1 overexpression promoted proliferation or migration.

Since ANO1 was identified to represent the endogenous CaCC currents, fast progress has been made on the function of ANO1 in the ICC (interstitial cells of Cajal) 34. The Farrugia group revealed that the intestinal ICC expressed ANO1 not only for slow wave generation (regulation of ICC cell excitability and intestinal movement rhythm) but also for ICC cell proliferation 24. They found that fewer proliferating ICC cells existed in an ANO1 knockout (ANO1) mouse. Application of Cl channel blockers (DIDS, Niflumic acid or Tamoxifen) decreased the proliferation of ANO1-expressing cells, ICC, and CFPAC-1 (pancreatic cancer-derived cell line) but had less effect on ANO1 ICC cells 24.

ANO1 proteins were highly expressed in prostate cancer tissues and metastatic prostate cancer cell lines, LNCaP and PC-3, and produced high density of CaCC currents. The Huang group discovered that knockdown of ANO1 in PC-3 cells resulted in a significant reduction of proliferation, metastasis, and invasion of the cells. Consistent with the results, intratumor injection of ANO1 shRNA to knockdown ANO1 completely inhibited the established growth of tumors derived from PC-3 xenografts in nude mice. The findings provided compelling evidence for the close correlation of ANO1 overexpression in prostate cancer tissues with their proliferation and metastasis. 11. The Bentires-Alj group produced the similar results with breast cancer cell lines and primary tumors that amplified and highly expressed ANO1 7. Likewise, knockdown of ANO1 in the ESCC cell lines, KYSE30 and KYSE510 cells significantly inhibited the proliferation of the cells 14.

In the following study, the Duvvuri group selected UM-SCC1 and T24 cell lines to evaluate whether the role of ANO1 in cancer cell proliferation is dependent on the 11q13 amplification. UM-SCC1 (a HNSCC cell line) harbors 11q13 amplification and overexpresses ANO1 while T24 (a bladder cancer cell line) does not. ANO1 was knocked down with lentiviral ANO1 shRNA in UM-SCC1 cells which were inoculated into nude mice. As result, the shRNA led to a significant decrease in xenograft tumor growth in vivo. While the T24 cell line was made overexpress ANO1 and used for inoculation, the xenograft growth was significantly enhanced 9, indicating that the cell proliferation mediated by ANO1 overexpression in T24 cells was independent of 11q13 amplification.

Another interesting investigation is worth mentioning. Using Ehrlich ascites cells, ANO1 or ANO6 were stably knocked down with siRNA for selection of clones expressing low levels of ANO1 or ANO6. ANO6 is another CaCC channel in the ANO family 35. Migration analysis showed that ANO1 knockdown clones changed their migrating direction, whereas ANO6 knockdown clones showed a reduced rate of cell migration by 40%. Therefore, the authors thought that ANO1 determined the direction while ANO6 the speed of cancer cell migration 10.

### ANO1 channel function

Research results have strongly suggested that the effect of ANO1 overexpression on the tumorigenic proliferation is probably exerted through its channel function. Since the ANO1-specific small molecule inhibitor, T16A_inh_-A01was found, the Cl channels could be differentiated and distinguished to selectively study the role and function of ANO1 channels 36. The effect of ANO1 on proliferation in ICC and in the ANO1-expressing CFPAC-1 was studied with T16A_inh_-A01. As a result, the inhibitor significantly inhibited CaCC currents and the proliferation of ICC in primary and organotypic cultures, and in the CFPAC-1 cell line. These data support the idea that ANO1 function is involved in the tumorigenic proliferation 12.

ANO1-K610A is a non-functional ANO1 mutant 37. In contrast to the wild type, overexpression of the ANO1 mutant in HEK293T, SCC1, and T24 cell lines did not promote anchorage-independent viability. To pursue the reasons for loss of the effect, the Duvvuri group found that the ANO1 mutant lost the ability to induce extracellular signal-regulated kinase (ERK)1/2 phosphorylation in the cell lines transfected with the ANO1 mutant. The activation of MAPK/ERK1/2 signaling pathway was demonstrated to enhance the growth of epithelial cancer cells, in particular, bladder cancer and HNSCC, which explains why the ANO1 mutant has lost the proliferating effect. Consistently, T16A_inh_-A01 abrogated tumor cell (UM-SCC1 and T24) proliferation in vitro 9. ANO1 channel function was also important for breast cancer cell viability 7.

***Viewpoint 2*****:** ANO1 stimulated cell migration rather than proliferation.

Using a HNSCC cell line, HEp-2, the Wasylyk group found that ANO1 overexpression by transfection of ANO1-expression vectors into HEp-2 cells stimulated cell movement, attachment, spreading, detachment, and invasion. Convincingly, downregulation of ANO1 expression by siRNA knockdown in SCC-25 (squamous cell carcinoma) cells that express a higher level of ANO1 than HEp-2 had the opposite effect. Inhibition of ANO1-channel activities with its inhibitors decreased the cell movement 33.

In line with this study, the Ruiz group reported that in the HNSCC cell lines, BHY and CAL-33, overexpression of ANO1 induced CaCC currents in cells and cell motility and migration in wound healing and in real time migration assays. BHY cells (expressing high levels of ANO1) migrated much faster than CAL-33 cells (expressing low levels of ANO1). Moreover, inhibition of ANO1 by its inhibitor, T16A_inh_-A01, strongly reduced migration of BHY cells. These results clearly indicate a role of ANO1 for cell migration 13. However, both groups surprisingly found that no matter whether ANO1 was knocked down or overexpressed in the SCC cells, their proliferation was not affected. It is unknown whether or not the phenomena were specific for these cell lines.

***Viewpoint 3*****:** ANO1 affected neither cellular proliferation nor migration.

Knockdown or inhibition of ANO1 via RNAi-mediated silencing or pharmacologic approach did not alter growth of the GIST-derived ANO1-overexpressing cell lines, GIST-T1 and GIST882 15. Transfection of functional ANO1-expressing vectors into HEK293 cells resulted in inducible overexpression of ANO1 proteins. However, the induced overexpression had no effect on either cellular proliferation or migration. The authors concluded that the resulting ANO1 channel activities were not directly involved in cell growth and motility 38. Nonetheless, ANO1 overexpression in HEK-293T which is derived from HEK293, contains SV40 T-antigen 39 and shares the cellular properties with HEK293, led to increased anchorage-independent proliferation 9. No interpretation is available for the results at present.

Against the idea that ANO1 promotes cell proliferation, ANO1 was recently recognized as a negative regulator of vascular smooth myocyte proliferation 40.

Overall, it seems that ANO1 overexpression produced different effects on the proliferation of different cancer cell lines, implying that the underlying process is complicated (Table 1).

## ANO1 Overexpression Degree is Associated With Tumor Malignancy

### HNSCC

The Ruiz group studied in detail how much ANO1 expression contributes to malignancy in HNSCC with 141 human HNSCC samples. They detected a highly significant correlation between 11q13 locus amplification and expression of ANO1 and showed that HNSCC patients with ANO1 overexpression had a poor overall survival. Correspondingly, lymph node metastases were more common in HNSCC patients with ANO1 protein positive than in those with ANO1 negative. *Because of the contribution of ANO1 to metastatic progression in HNSCC, poor survival, or tumor malignancy in HNSCC patients is correlated with the presence of ANO1* 13. Shi et al. obtained similar results 14.

Another contribution by the Ruiz group is that from more than 3000 human samples including 80 different tumor types, the authors found that apart from HNSCC and GISTs, ANO1 protein was rarely expressed in other tumor samples or normal human tissues 13. Unfortunately, the results were obtained from IHC only. Further confirmation is required with independent methods, for example, Western blotting to detect the presence of ANO1 protein.

To test the possible role of ANO1 in malignant transformation and to determine the association between ANO1 overexpression and clinical outcome of patients with HNSCC, the Duvvuri group made a similar investigation. However, *they found that ˜85% of HNSCC tumors were ANO1 positive*. Kaplan–Meier survival analysis showed that patients with high-level tumor expression of ANO1 had decreased overall survival 9. It is noteworthy that the Ruiz group showed that only ∼10% HNSCC tumors were ANO1 positive 13. That is why we suggest that the percentage number ought to be confirmed with more sensitive methods.

### Prostate cancer

With the human pathologic tissue specimens from patients (classified by TNM staging) and human tissue arrays by IHC with specific antibody against ANO1, the expression of ANO1 was found to be closely correlated with the malignancy of the prostate cancers. The study reached a conclusion: the higher the expression of ANO1, the higher the malignancy of the prostate cancer 11.

### Breast cancer

The Bentires-Alj group found that ANO1 is amplified and highly expressed in breast cancer cell lines and primary tumors. The 11q13 region is amplified in ∼15% of breast cancers. Amplification of ANO1 in breast cancer was correlated with disease grade and poor prognosis 7.

Using the Oncomine database to determine the expression of ANO1 in normal and malignant tissues from a variety of tumor types, the Duvvuri group found that although ANO1 may be expressed at a high level in normal breast tissue, its expression is even higher in neoplastic breast tissue. This suggests that although endogenous ANO1 expression may be high in some normal tissues, malignant cells derived from these tissues further upregulate ANO1 expression, implicating that ANO1 may be a potential target gene in malignant transformation 9.

## Potential Mechanisms of ANO1 Overexpression in Cell Proliferation and Tumorigenesis

The above reports suggest that only if ANO1 has channel function, can it exert positive effect on the cell proliferation, migration, and tumorigenesis. However, how ANO1 channel function is involved in these processes remains elusive. Duvvuri et al. found that ANO1 overexpression-induced cancer cell proliferation and tumor growth were accompanied with increase in extracellular signal-regulated kinase (ERK)1/2 activation and CCND1 induction. Further experiments demonstrated that overexpression of ANO1 in T24 and UM-SCC1 cancer cells induced RAS-RAF-MEK-ERK pathway activation 9.

**Figure 1:**
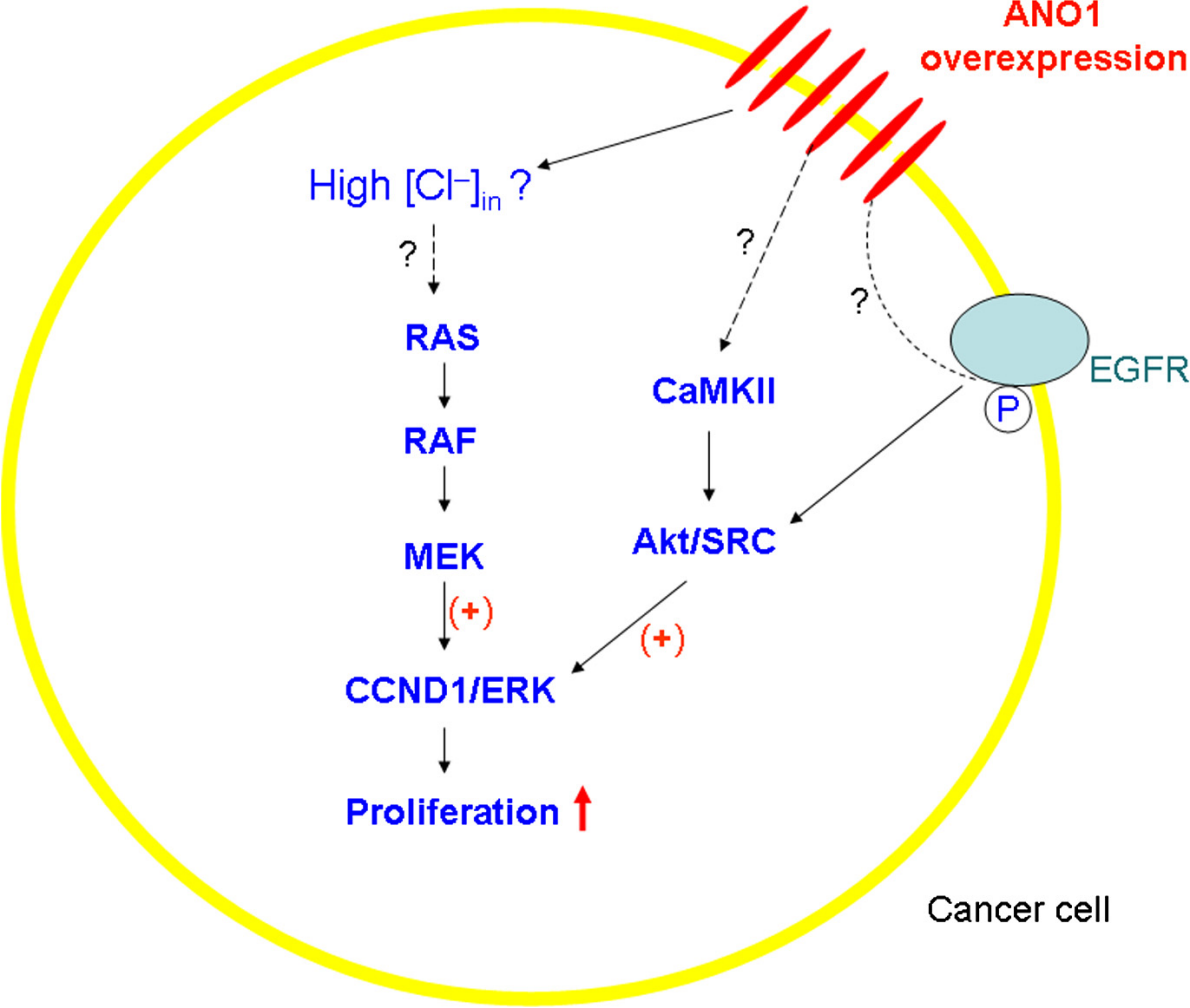
ANO1 overexpression enhances cancerous cell proliferation through signaling pathways. See text for explanation.

Pharmacologic inhibition of MEK/ERK (using specific inhibitors for MAPK/ERK signaling pathway, UO126 and AZD6244) and genetic inactivation of ERK1/2 (using siRNA and dominant-negative constructs) abrogated the growth effect of ANO1, indicating a role for MAPK activation to enhance the ANO1-mediated proliferation. The data from the nonfunctional mutant ANO1-K610A raised the possibility that the overexpressed ANO1 with channel function affected the ERK activation by modulating [Cl^−^]_i_ of cancer cells 9 (Fig. 1).

**Figure 2:**
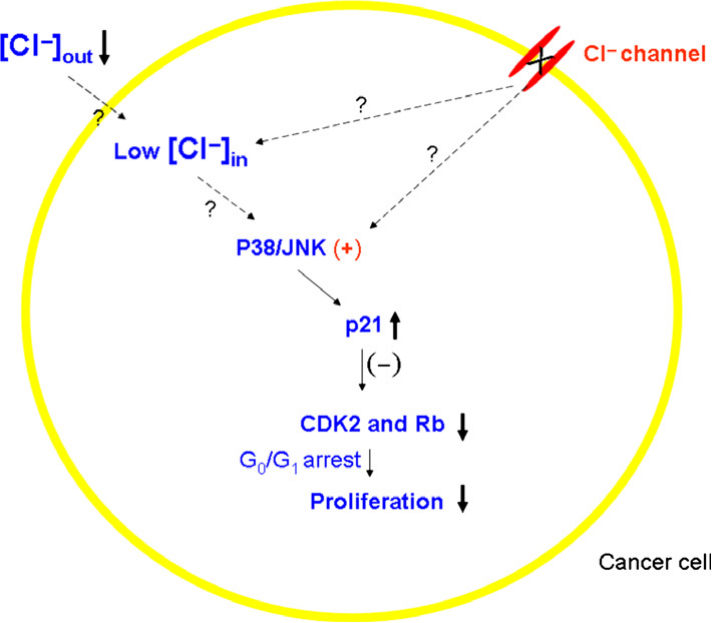
Inhibited Cl channels and low intracellular [Cl^−^] suppress cancerous cell proliferation through signaling pathways. See text for explanation.

About two decades ago, anion channels were found to be associated with cellular proliferation and tumorigenesis 41. Now, the relevant Cl^−^ channels have extended to CaCC (e.g., ANO1, bestrophin1, and hCLCA2) 42,43,24,44, voltage-dependent Cl channels (e.g., CLC3) 45–49 and VRAC 50–52,41. The studies put forward two possible mechanisms by which the Cl^**−**^ channels may be involved in the regulation of cell proliferation (Fig. 2). One is the Cl channel molecule itself. The active channels may affect cellular signaling pathways through some domains on the channels 53, or their intracellular domains may have some enzyme activities, for example, kinases 54. Compelling evidence has not been found for the possibility. The other one is the intracellular Cl^**−**^ concentrations ([Cl^−^]_i_). The changes of [Cl^−^]_i_ induced by the Cl^−^ channel activities may affect the cellular proliferation.

[Cl^−^]_i_ has recently been considered as a contributor to the tumorigenesis 55,56. The Marunaka group cultured the gastric cancer cell line, MKN28 with low [Cl^−^] medium, which lowered [Cl^−^]_i_ and found that the cell proliferation was significantly reduced with the cell cycle arrested at the G(0)/G(1) phase. The arrest was caused by diminution of phosphorylated cdc2/CDK2 and Rb, key accelerators of transition from G(1) to S phase and G(2) to M phase in the cell cycle. The later study demonstrated that the low [Cl^−^]_i_ upregulated p21 (a CDK inhibitor), implying that inhibition of CDK2 and Rb was caused by the upregulated p21 57. Now the group has clarified that activation of mitogen-activated protein kinase (MAPKs including p38 and JNK) by low [Cl^−^]_i_ led to the upregulation of p21 58,59 (Fig. 2). *Here, the Marunaka group argued that low [Cl]*_*i*_ *activated MAPK, which suppressed proliferation*.

At the moment, one may ask: does the overexpressed ANO1 increase or reduce the [Cl^−^]_i_ in cancer cell lines? Unfortunately, no evidence is available to answer the question for now. Logically, it fits into the picture by the Marunaka group if the [Cl^−^]_i_ is elevated by the overexpressed ANO1, which promotes the cellular proliferation. However, it has been reported that [Ca^2+^]_i_ is lower in cancer cells 60,61. Therefore, it is hard to estimate how much the overexpressed ANO1 is activated there and it remains an enigma how the activated ANO1 channels promote cellular proliferation.

Another noteworthy aspect is that MAPK activation suppressed proliferation as shown in Figure 2 while it was opposite in Figure 1. Therefore, it seems that different MAPK members play different roles in cellular proliferation. The studies have left a question to answer: *Does MAPK activity depend on [Cl*^*−*^*]*_*i*_*?* If intracellular Cl^−^ anions determine or affect the activities of signaling factors, we think that the first experiment to test the effect of [Cl^−^] on the activities of the factors (e.g., phosphorylation of ERK1/2) must be done in tubes to justify the hypothesis.

As to the mechanism how ANO1 regulates proliferation, Britschgi et al. discovered that EGF receptor (EGFR) and calmodulin-dependent protein kinase II (CaMKII) were also involved (Fig. 1). EGFR and CaMKII signalings regulate the ERK activation through Akt, v-src sarcoma viral oncogene homolog (SRC) in vitro and in vivo while ANO1 knockdown or pharmacological inhibition of its Cl^−^ channel activity resulted in downregulation of the whole signaling pathway 7.

Following these discoveries, more specific questions ensue about how the activated ANO1 acts on the signaling factors: *Does ANO1 affect the signaling pathways through regulating the [Cl]*_*i*_ *or work as a signaling factor only when it is activated? Or does the activated ANO1 have any active domain(s) which can stimulate signaling factors?*

Another finding from the Bauer group turns out to be very interesting as well 15. Since the silencing knockdown of DOG1 delayed the growth of GIST xenografts in vivo, analysis of expression profiling of explanted GIST tumors after DOG1 knockdown revealed a strong upregulation in the expression of insulin-like growth factor-binding protein 5 (IGFBP5), which is a potent antiangiogenic factor and a tumor suppressor. Similar results were obtained from DOG1-negative cells (GIST430B) where the IGFBP5 mRNA transcripts were 5000-fold higher than that in the parental DOG1-positive cells (GIST430). The result implied that DOG1 silencing may have delayed the growth of GIST xenografts through IGFBP5 upregulation which may have inhibited the angiogenesis via IGF pathway 15. How ANO1 channel silencing regulates IGFBP5 expression is an enigmatic and attractive issue.

Overall, there is no strong evidence as yet to interpret how an activated ANO1 affects cancer cell proliferation and migration.

## What Causes the Overexpression of ANO1 in the Cancer Tissues?

How ANO1 overexpression (e.g., at the level of transcription or posttranscription) is triggered by signaling pathways or transcription factors during the tumorigenesis remains totally unknown.

DOG1 (ANO1) overexpression was observed not only in the tumors with 11q13 amplification but also in a large fraction of the tumors without the 11q13 amplification. Similar phenomena also were observed in the oral squamous cell carcinomas (OSCC) cell lines, suggesting that the overexpression of the *DOG1* gene in the amplicon core was not an artifact of the cancer cell cultures, and the 11q13 amplification is not the only means to achieve the overexpression of the *DOG1* gene in the tumorigenesis 32. *It implies that ANO1 overexpression in tumorigenesis is not contingent on the 11q13 amplification but may have its own regulatory mechanism*. Supporting this argument, the Duvvuri group, with T24 (a bladder cancer cell line) which does not harbor the 11q13 amplification, also suggested that the role of ANO1 in proliferation may not require the expression of other genes within the 11q13 amplified core region 9.

It is well known that some signaling pathways are turned off but others turned on in the developmental process. Some mechanisms faded in adults may have been reactivated in the proliferating cancer cells. The returned signaling factors eventually cause overexpression of proliferation-relating proteins including ANO1. A famous signaling pathway is Wnt. Since high-expression level of ANO1 occurs in the breast cancer, we tested several Wnt pathways to see which one may be correlated with the ANO1 overexpression. We found that ANO1 expression in breast cancer cell line, MCF7 is dramatically upregulated by some Wnt signaling factors (unpubl. data).

## Inhibition of ANO1 May Be a Potential Target for Cancer Therapy

There are at least two ways to explain the relation of ANO1 overexpression with tumorigenesis. One is that ANO1 overexpression is a causal factor for the tumorigenesis. If ANO1 functions as an oncogene, when it is overexpressed, it will participate in the initiation of the process of the tumorigenesis. The other one is that the overexpression of ANO1 may be an end step in tumorigenesis. In either case, block of the channel ought to interrupt the tumorigenesis. Especially, if the factors upstream of ANO1 transcription can be revealed, inhibition of the factors will be more helpful to resist the overexpression of ANO1 and help stop the tumorigenic process. Since ANO1-specific blocker reduced the proliferation of cultured cancer cells (CFPAC-1, SCC1, T24, etc.) 7,9,12, development of more specific and more potent ANO1 inhibitors will be useful for cancer therapy. A new ANO1 blocker, MONNA with much more potency has been developed and its effect as a cancer treatment is greatly anticipated 62.
